# Combined large cell neuroendocrine carcinoma, lung adenocarcinoma, and squamous cell carcinoma: a case report and review of the literature

**DOI:** 10.1186/s13019-023-02349-4

**Published:** 2023-08-31

**Authors:** Zirui Zhu, You Liu, Hengliang Xu, Haoyong Ning, Yanmin Xia, Leilei Shen

**Affiliations:** 1 Department of Thoracic Surgery, Hainan hospital of PLA general hospital, Sanya, 572013 PR China; 2 Department of Pathology, Hainan hospital of PLA general hospital, Sanya, PR China

**Keywords:** LCNEC, Combined tumor, Adenocarcinoma, Squamous cell carcinoma, Adjuvant chemotherapy

## Abstract

**Background:**

Combined large cell neuroendocrine carcinoma (C-LCNEC) has a poor prognosis and there is no consensus about the treatment regimen for both LCNEC and C-LCNEC patients.

**Case presentation:**

The patient was a 47-year-old female who received surgical resection. The postoperative histology and staging of the tumor suggested C-LCNEC with adenocarcinoma and squamous cell carcinoma and T2aN0M0 stage IB. Next-generation sequencing test showed *KIF5B/RET* fusion mutation without *EGFR*, *ALK*, *RB1*, and *TP53* alterations. Adjuvant chemotherapy with 4-cycle docetaxel plus carboplatin was given and brain metastasis occurred after 10 months.

**Conclusions:**

C-LCNEC with adenocarcinoma and squamous cell carcinoma is rare and highly aggressive cancer. Surgical resection and adjuvant chemotherapy with SCLC regimen may improve the disease-free survival and overall survival. The accumulation of similar cases will clarify the profile and management of the disease.

## Background

The 2021 World Health Organization (WHO) classification of lung tumors states that lung combined tumors generally occur in the setting of high-grade carcinomas and not in the carcinoid [1]. Combined large cell neuroendocrine carcinoma (C-LCNEC) consists of LCNEC and adenocarcinoma (ADC), squamous cell carcinoma (SCC) or small cell lung cancer (SCLC), which accounting for more than 10% of LCNEC patients. To our knowledge, LCNEC accounts for approximately 3% of all lung cancers and possesses aggressive trait [2]. Previous studies have demonstrated that C-LCNEC was more aggressive than LCNEC [3]. LCNEC/ADC and LCNEC/SCC are relatively common in prior studies and the comparison of their clinicopathological features and prognosis have been reported [2–8]. Here, we present an interesting patient with combined large cell neuroendocrine carcinoma, lung adenocarcinoma, and squamous cell carcinoma to improve our understanding of the diversity of the disease.

## Case presentation

**Fig. 1:**
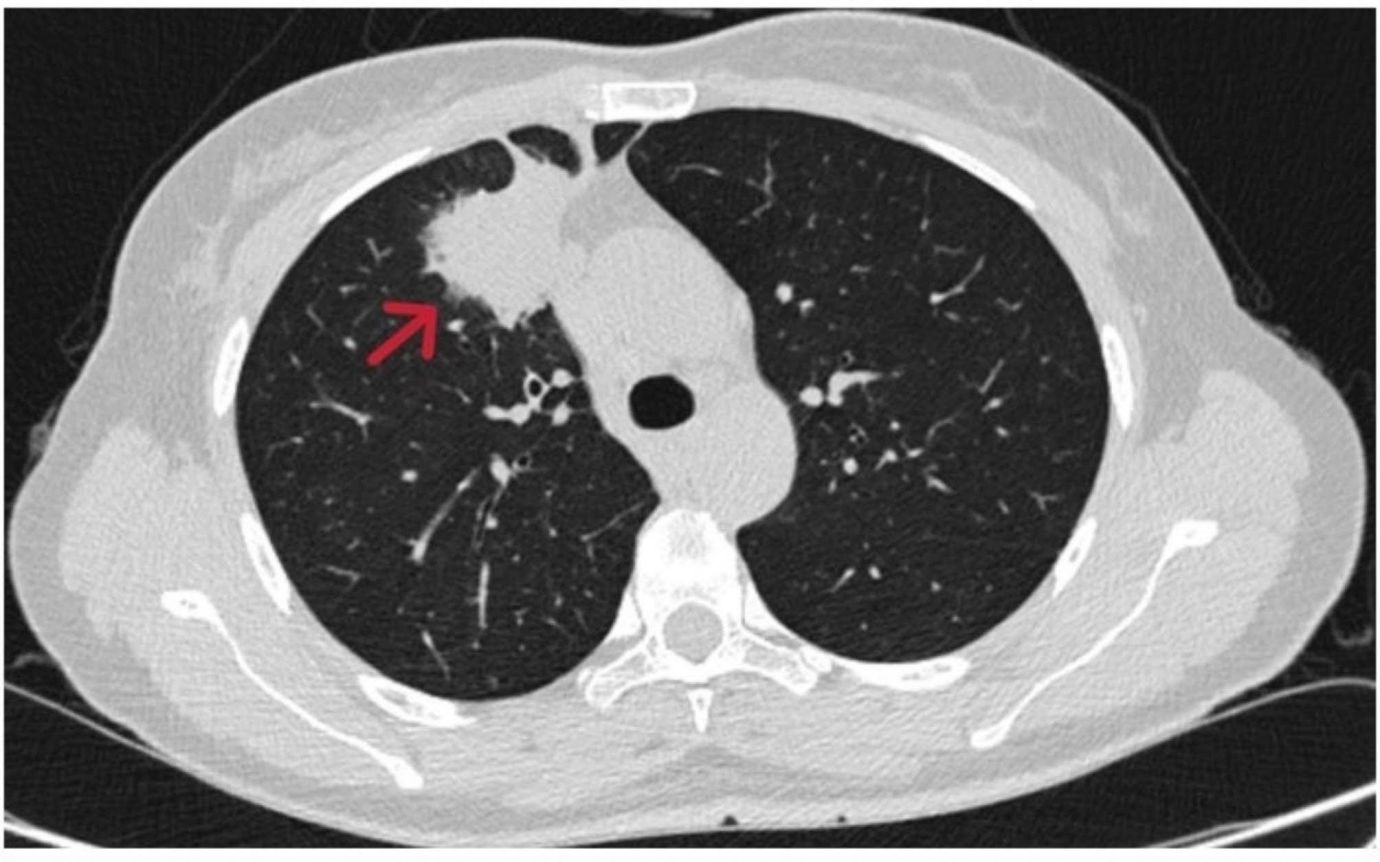
CT showed a 36.7mm *34.1mm mass with irregular shape, lobalation, spiculation, pleural indentation, and vessel convergence

**Fig. 2:**
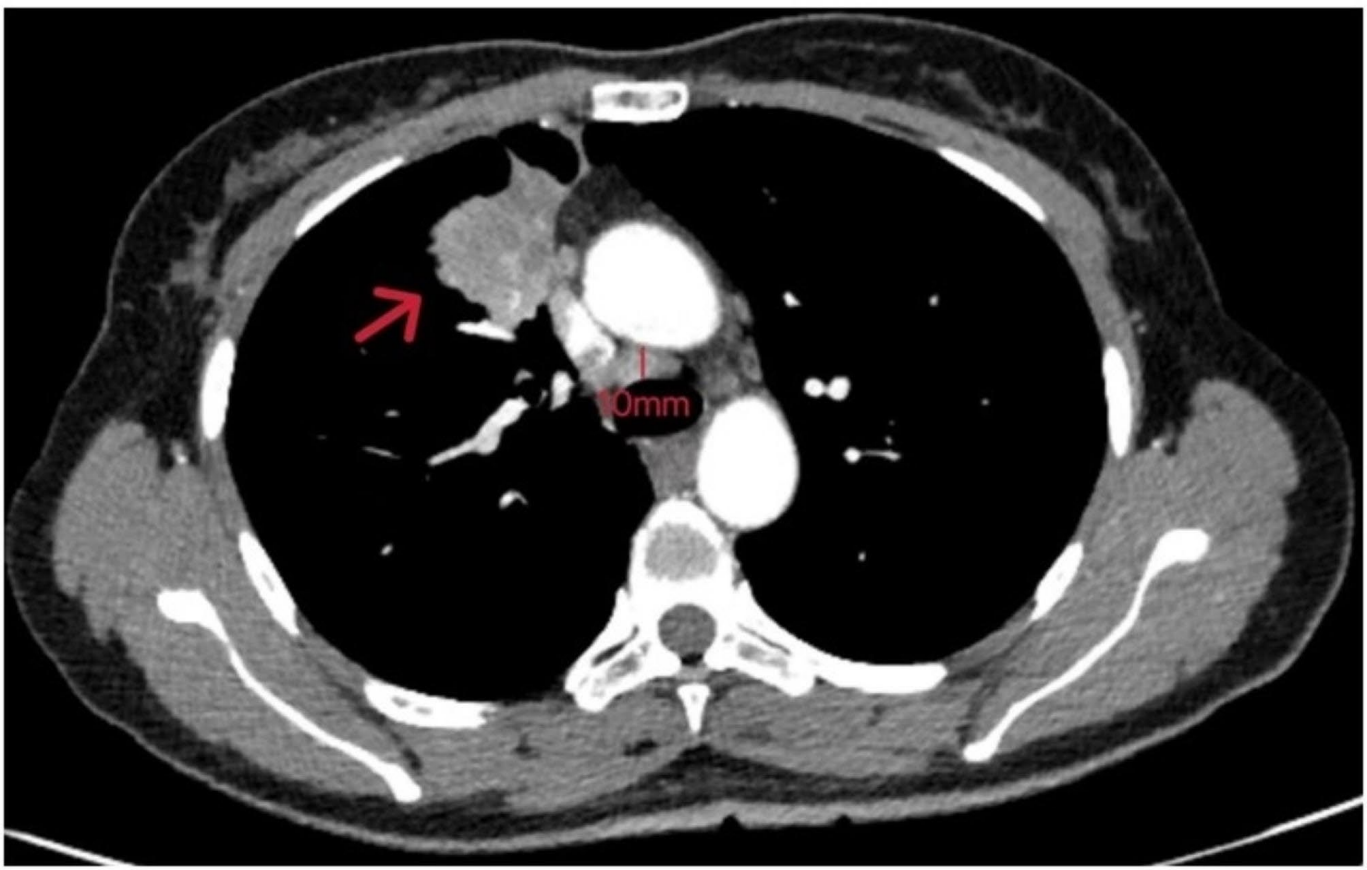
Enhanced contrast CT elucidated that statin 4R lymph node was slightly enlarged with 10mm short axis

**Fig. 3:**
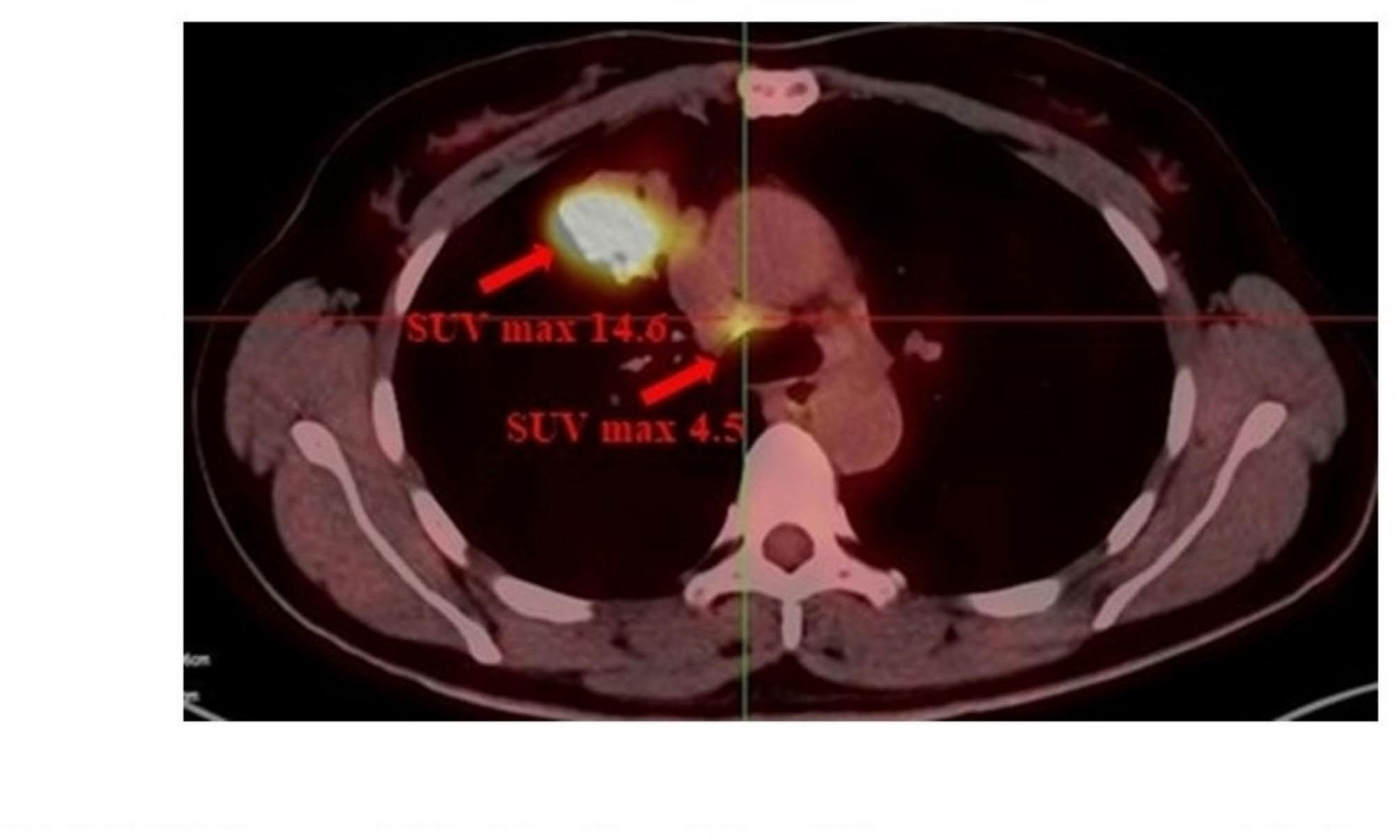
PET-CT suggested that the 40mm*39mm*34mm mass was suspected of lung cancer with SUV max 14.6 and the swollen mediastinal lymph node 4R with approximately 10mm short axis had a high uptake (SUV max = 4.5)

**Fig. 4:**
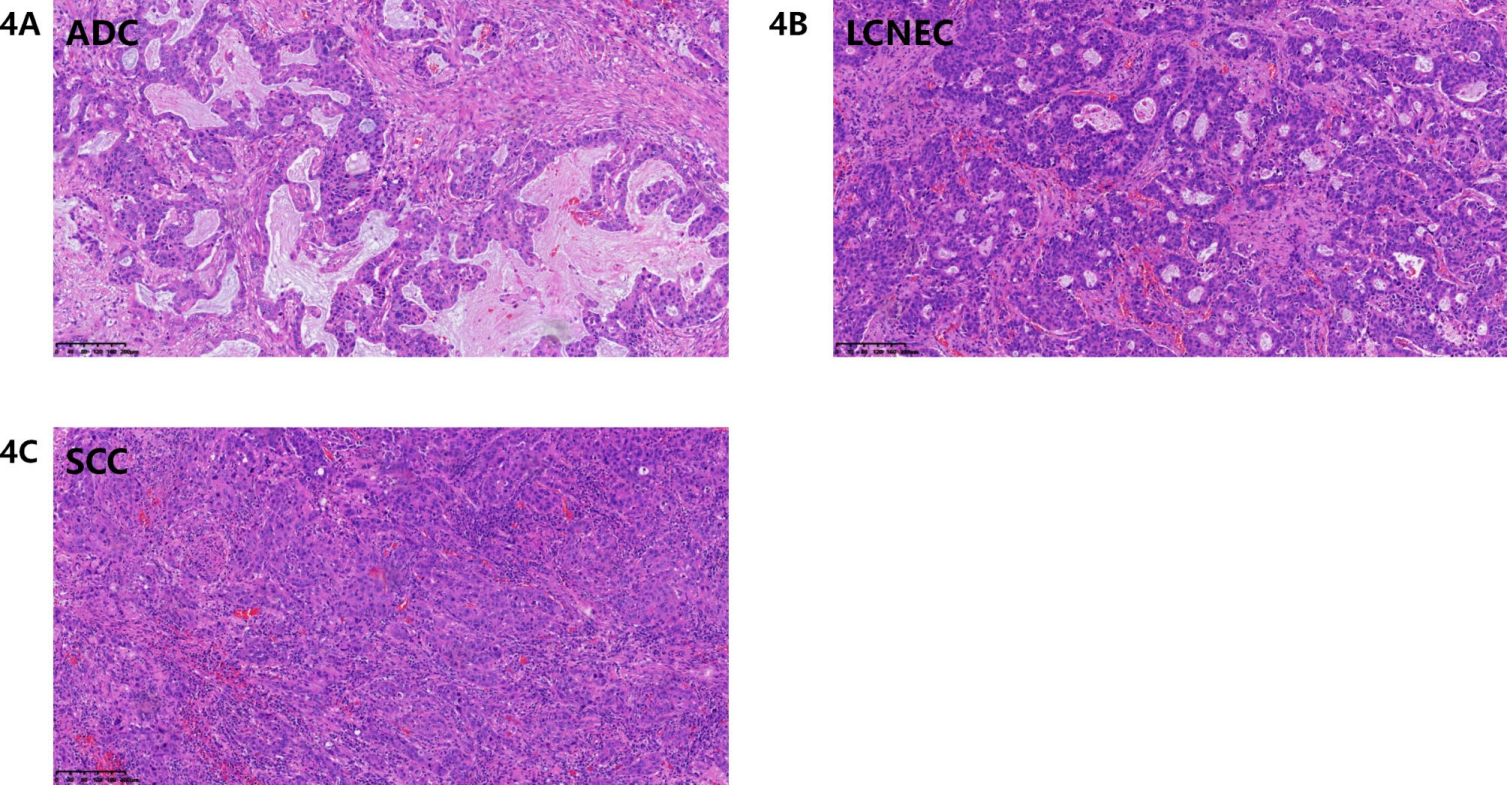
HE staining (x10) showed the tumor consisted of 40% of acinar adenocarcinoma **(A)**, 10% of mucous adenocarcinoma, 40% of LCNEC (B), and 10% of poor cell differentiated SCC (C)

**Fig. 5:**
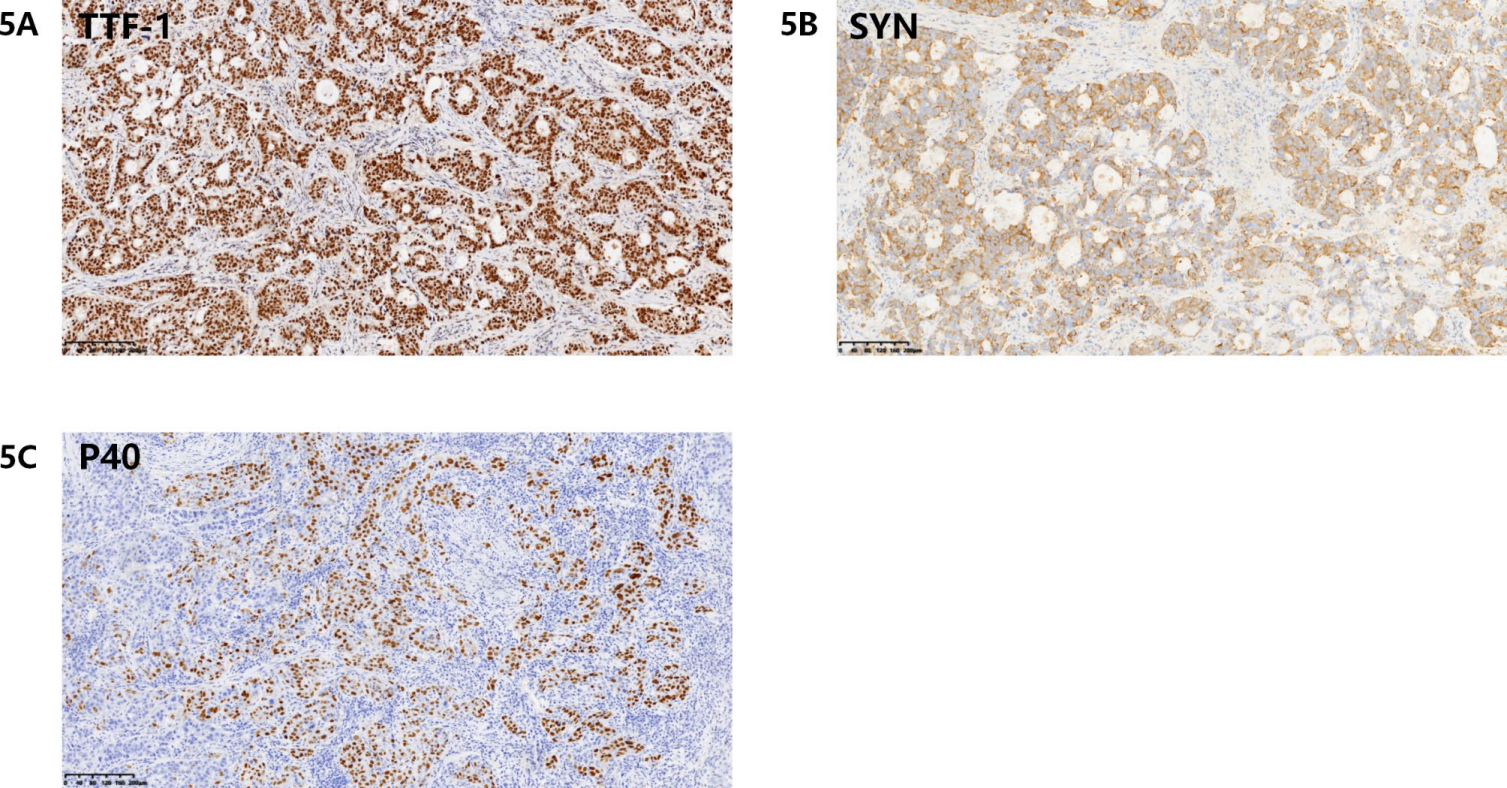
The immunohistochemical profile (x10) showed that ADC was positive for TTF-1 **(A)**, LCNEC was positive for synaptophysin **(B)**, and SCC was positive for p40 **(C)**

**Fig. 6:**
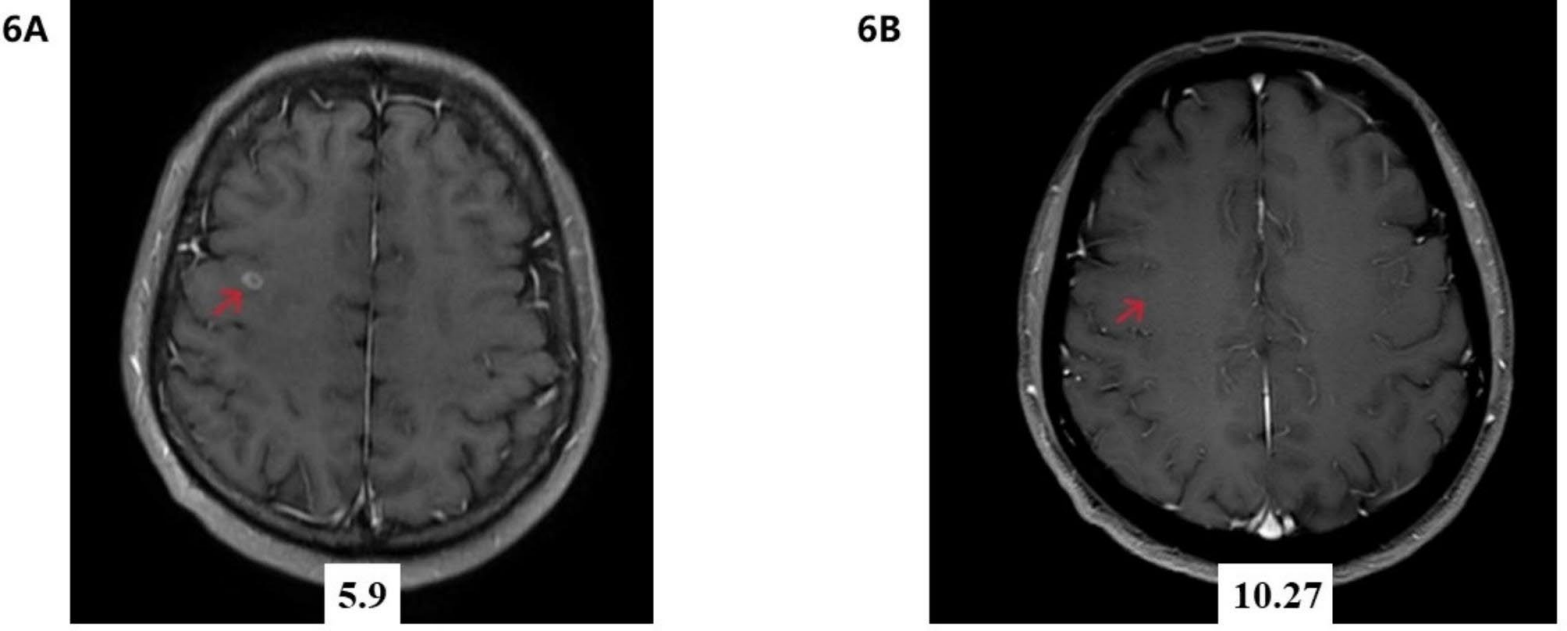
Brain MRI found that abnormal signal in the right frontallobe on May 9th **(A)**, and small metastases has decreased on October 27th **(B)**

A 47-year-old woman found abnormality of carcinoembryonic antigen (CEA) 14.18 ug/L without positive gastrointestinal endoscope findings in October 2020. Chest plain computed tomography (CT) was conducted until 3 months later with double-check high CEA (17.18ug/L), and the CT revealed a 26.7 mm plus 24.1 mm solid nodule in the right upper lobe. She had no smoking history and family history of lung cancer. The patient received CT examination on June 7th 2021 after anti-infective therapy, which showed a 36.7 mm plus 34.1 mm mass with irregular shape, lobalation, spiculation, pleural indentation, and vessel convergence (Fig. 1). Enhanced contrast CT elucidated that station 4R lymph node was slightly enlarged with 10 mm short axis (Fig. 2). The CEA was 26.96 ug/L, squamous cell carcinoma antigen was 1.98 ng/mL, CYFRA 21 − 1 was 3.12 ng/mL, and pro-gastric releasing peptide was 65 ng/L. Following positron emission tomography-computed tomography (PET-CT) suggested that the 40 mm*39mm*34mm mass was highly suspected of lung cancer with standard uptake value (SUV) 14.6 (Fig. 3). And the swollen mediastinal lymph node 3 A and 4R had a high uptake (SUV max = 4.5) (Fig. 3). Brain magnetic resonance imaging (MRI) and bone scan had negative results. The CT guided pulmonary biopsy was performed and the pathology suggested poor cell differentiated lung adenocarcinoma with neuroendocrinization. The patient received right upper lobectomy with systemic lymph node dissection. Postoperative pathological analysis confirmed the diagnosis of combined LCNEC, ADC, and SCC. Grossly, the tumor was solid, gray-white, with a moderate hardness texture and vague boundaries. The size was 40 mm*35mm*25mmwithout visceral pleural invasion. The lymph nodes including station 2, 3, 4, 7, 10, and 11 were all negative. Histology showed that the tumor consisted of 40% acinar adenocarcinoma (Fig. 4A), 10% mucinous adenocarcinoma, 40% LCNEC (Fig. 4B), and 10% poor cell differentiated SCC (Fig. 4C). The immunohistochemical profile revealed that ADC cells were positive for napsin-A and thyroid transcription factor 1(TTF-1) (Fig. 5A), LCNEC cells was positive for synaptophysin (Fig. 5B), and SCC cells was positive for p63 and p40 (Fig. 5C). The patient was diagnosed with pathological T2aN0M0 stage IB, and next-generation sequencing (NGS) test showed that KIF5B/RET fusion mutation was observed in the entire paraffin section with LCNEC, SCC, and ADC components. Meanwile, *EGFR*, *ALK*, *ROS1*, *KRAS*, *BRAF-V600E*, *ERBB2*, *MET*, *NTRK*, *RB1*and *TP53* alterations were not detected. Four cycles (21 days per cycle) of docetaxel (100 mg) plus carboplatin (500 mg) were conducted without obvious grade>3 adverse events. No relapse or metastatic signs were observed after 4 cycles in October 2021. Regular follow-up was requested until on May 9th 2022 the brain MRI found that multiple abnormal signals in the right frontal lobe (Fig. 6A), which were considered as metastatic tumors. Bone scan and CT of chest and abdominal revealed no positive findings and tumor biomarkers including CEA, squamous cell carcinoma antigen (SCC), and neuron specific enolase (NSE) were normal. The patient had a recurrence and was staged as advanced. The patient received brain radiotherapy (40 Gy/10F) through HyperArc without targeted therapy or chemotherapy and multiple small metastases in the brain has decreased than before on October 27th (Fig. 6B), which was evaluated as stable disease.

**Table:** Effective markers for each cancer type in immunohistochemistry

Cancer type	Effective marker
NET	CgA, Syn, CD56
SCC	P40, p63, CK5/6
ADC	TTF−1, Napsin A, CK7

Abbreviations: NET, neuroendocrine tumor; SCC, squamous cell carcinoma; ADC, adenocarcinoma; CgA, chromogramin A; Syn, synaptophysin; TTF-1, thyroid tran

## Discussion

It is well-known that pulmonary LCNEC is a highly aggressive and rare form of cancer with high relapse rate and poor prognosis. Prior publications had reported that the proportion of C-LCNEC varied between 10% and 49%, and LCNEC/ADC was the frequent type [4, 7, 8]. Yang ZY et al. reported 74% (71/96) of LCNEC/ADC in their 96 patients’ cohort study, and LCNEC/SCC was more likely to occur in male, elderly, heavy smoker [4]. In Zhang JT et al.’s cohort study, there were 21 LCNEC/ADC patients out of 30 LCNEC patients [3], and LCNEC/ADC accounted for 56% (28/50) in Grand B et al.’s study [8].

The diagnostic criterion of LCNEC was first reported in 1991 by Travis et al. [9] and was classified into neuroendocrine tumors category in the 1999 edition WHO classification and subsequent 2004, 2015, and 2021 edition. However, whether LCNEC should be treated as small cell lung cancer (SCLC) or non-small cell lung cancer (NSCLC) remains controversial. Several studies failed to draw consistent conclusions due to the relatively small sample size and retrospective nature [2–4, 6, 8, 10, 11]. In this case, patient was assessed stage IB preoperative, and surgical resection with LND was obviously the most effective treatment [4]. The postoperative strategy is debatable and nonuniform. Prior studies have demonstrated that sage II or higher LCNEC patients could benefit from adjuvant chemotherapy, whereas stage I patients showed indistinctive benefit [4, 12]. Some other studies draw the opposite conclusions that even early-stage I patients could benefit from postoperative chemotherapy [13, 14]. As described before, C-LCNEC has poorer survival than pure LCNEC and may benefit more from adjuvant chemotherapy or other treatments. However, in Yang ZY et al.’s cohort study, stage I patients with C-LCNEC could not benefit from chemotherapy regardless of LCNEC/ADC or LCNEC/SCC, while obvious benefit was observed in stage II-III patients [4]. The case reported here received 4-cycle adjuvant chemotherapy and the DFS was 10 months. The neuroendocrine nature of the tumor and preoperatively positive CEA may contribute to the final relapse. As we all acknowledge that positive CEA is associated with poor DFS and OS in LCNEC [6]. She could have consulted further at the first time when her serum CEA was abnormal.

The regimen of postoperative chemotherapy is another controversy. Prior publications have suggested that RB1 + TP53 alterations are effective biomarkers for subclassification of LCNEC and may guide precise adjuvant chemotherapy for different subgroup patients [15–17]. Two studies classified LCNEC into 2 major subgroups: SCLC-like LCNEC patients were those with RB1 and TP53 mutations or copy-number loss, and NSCLC-like LCNEC patients had no co-alterations of RB1 + TP53 [15, 16]. Zhuo M et al. demonstrated that SCLC-like LCNEC patients treated with pemetrexed-platinum and gemcitabine/taxane-platinum had lower objective response rate (0% vs. 75%, *P* = 0.02), progression-free survival (2.4 months vs. 8.3months, *P* = 0.002) and overall survival (4.1 months vs. 9.7 months, *P* = 0.600) compared to etoposide-platinum regimens, whereas NSCLC-like LCNEC patients treated with etoposide-platinum or pemetrexed-platinum was associated with superior overall survival (19.6 months vs. 9.4 months, *P* = 0.07) compared to gemcitabine/taxane-platinum doublets [15]. Shen YC et al. also presented that etoposide-platinum doublets acted as an independent prognostic factor for OS [6]. Rekhtman et al. suggested that both NSCLC-like and SCLC-like LCNEC patients may be sensitive to PD-1/PD-L1 immune checkpoint inhibitors [16]. Patient in our case was categorized into NSCLC-like group for lack of RB1 and TP53 mutations, whereas KIF5B/RET fusion mutation was detected. Recent study confirmed the findings that patients who received SCLC regimen had better DFS and OS than patients who received NSCLC regimen in both pure LCNEC (*P* = 0.015 and *P* = 0.033, respectively) and C-LCNEC patients (*P* = 0.011 and *P* = 0.010, respectively) [4]. Meanwhile, differences regarding DFS and OS between the LCNEC/ADC group and the LCNEC/SCC group were not significant. Though without consensus about what treatment should be given to patients with C-LCNEC, this publication has suggested that C-LCNEC patients should follow neuroendocrine carcinoma’s strategy. Combined LCNEC/ADC /SCC was first reported in 2015 by Tenjin et al. [18]. Four cycles of adjuvant regimen with cisplatin and irinotecan were given the pathological T1N1M0 stage IIA patient, and one-year DFS was achieved. To the best of our knowledge, the case we presented was the second study and docetaxel plus carboplatin was given, which achieved a 10-month DFS time and was shorter than the first reported study [19]. This might verify the finding that patients who received SCLC regimen had better DFS than patients who received NSCLC regimen again.

Few studies also discovered the scarce *EGFR* mutations and *ALK* rearrangement rates in some C-LCNEC patients (8.33%, and 5.77%, respectively) [6]. Application of targeted therapy in both LCNEC and C-LCNEC patients is disputable for lack of evidence. *KIF5B/RET* fusion mutation was observed in our report and pralsetinib is an alternative. Back to the histopathology of this case, TTF-1 and P40 are immunohistochemical markers of the first choice to detect adenocarcinomatous and squamous cell differentiation. Though with the coexistence of LCNEC and SCC, the tumor is basically adenocarcinoma with *KIF5B/RET* fusion. However, the studies about the prognosis and therapy were few.

In summary, combined large cell neuroendocrine carcinoma with adenocarcinoma and squamous cell carcinoma is rare and highly aggressive cancer with poor prognosis. Surgical resection and adjuvant chemotherapy with SCLC regimen may improve the DFS and OS. The accumulation of similar cases will clarify the profile and management of the disease.

**Table:** 

Authors	Year of publication	Groups	Treatment	Results
Yang ZY, et al. [4]	2022; Article	LCNEC/AD (71); LCNEC/SCC (25)	NSCLC-regimen (43) vs. SCLC-regimen (35)	SCLC regimen had longer DFS and OS (P = 0.011 and P = 0.010).
Zhang JT, et al. [3]	2020; Article	Pure LCNEC (220); combined LCNEC (30)	NSCLC-regimen vs. SCLC-regimen	No significance between different treatments for adjuvant modality (P = 0.112).
Oda Risa, et al. [20]	2020; Case report	Combined LCNEC/SCC	Surgery alone	8 months DFS.
Zhuo ML, et al. [15]	2020; Article	SCLC -like LCNEC (15); NSCLC-like LCNEC (48)	SCLC-regimen vs. NSCLC-regimen	SCLC regimen had higher disease control rate (P = 0.007), response rate (P = 0.02), and longer PFS (P = 0.002).
Shen YC, et al. [6]	2020; Article	LCNEC	etoposide–platinum regimen (21); pemetrexed/cisplatin (26); gemcitabine/vinorelbine/paclitaxel-platinum (28)	etoposide–platinum regimen had longer median DFS.

## Data Availability

Please contact the corresponding author to request this information.
